# Contribution of serum anti-Müllerian hormone in the management of azoospermia and the prediction of testicular sperm retrieval outcomes: a study of 155 adult men

**DOI:** 10.1186/s12610-021-00133-9

**Published:** 2021-06-17

**Authors:** Hamza Benderradji, Julie Prasivoravong, François Marcelli, Anne-Laure Barbotin, Sophie Catteau-Jonard, Carole Marchetti, Catherine Guittard, Philippe Puech, Valérie Mitchell, Jean-Marc Rigot, Arnauld Villers, Pascal Pigny, Clara Leroy

**Affiliations:** 1 Department of Andrology, Urology and Renal Transplantation, Claude Huriez Hospital, Lille University Hospital, 1 Place de Verdun, 59045 Lille Cedex, France; 2 Department of Reproductive Biology-Spermiology-CECOS, Lille University Hospital, Lille, France; 3 Department of Endocrine Gynecology and Reproductive Medicine, Lille University Hospital, Lille, France; 4 Department of Reproductive Biology, BIOLILLE Laboratory, Lille, France; 5 Department of Reproductive Medicine, Bois Blanc Hospital, Lille, France; 6 Department of Radiology, Lille University Hospital, Lille, France; 7 Department of Biochemistry & Hormonology, Lille University Hospital, Lille, France

**Keywords:** Anti-Müllerian hormone, Obstructive and non-obstructive azoospermia, Testicular sperm retrieval, Spermatogenesis, Klinefelter syndrome, Hormone anti-Müllerienne, Azoospermie obstructive et non obstructive, Extraction chirurgicale de spermatozoïdes testiculaires, Spermatogénèse, Syndrome de Klinefelter

## Abstract

**Background:**

Testicular sperm extraction (TESE) is the method of choice for recovering spermatozoa in patients with azoospermia. However, the lack of reliable biomarkers makes it impossible to predict sperm retrieval outcomes at TESE. To date, little attention has been given to anti-Müllerian hormone (AMH) serum levels in adult men with altered spermatogenesis. In this study we aimed to investigate whether serum concentrations of AMH and the AMH to total testosterone ratio (AMH/T) might be predictive factors for sperm retrieval outcomes during TESE in a cohort of 155 adult Caucasian men with azoospermia.

**Results:**

AMH serum levels were significantly lower in nonobstructive azoospermia (NOA) that was unexplained, cryptorchidism-related, cytotoxic and genetic (medians [pmol/l] = 30.1; 21.8; 26.7; 7.3; and *p* = 0.02; 0.001; 0.04; <0.0001, respectively]) compared with obstructive azoospermia (OA) (median = 44.8 pmol/l). Lowest values were observed in cases of genetic NOA (*p* < 0.0001, compared with unexplained NOA) and especially in individuals with non-mosaic Klinefelter syndrome (median = 2.3 pmol/l, *p* <0.0001). Medians of AMH/T values were significantly lower in genetic NOA compared to unexplained, cryptorchidism-related NOA as well as OA. Only serum concentrations of AMH differed significantly between positive and negative groups in men with non-mosaic Klinefelter syndrome. The optimal cut-off of serum AMH was set at 2.5 pmol/l. The sensitivity, specificity, positive predictive value, negative predictive value and accuracy of this cut-off to predict negative outcomes of SR were 100 %, 76.9 %, 66.6 %, 100 and 84.2 %, respectively.

**Conclusions:**

Serum AMH levels, but not AMH/T values, are a good marker for Sertoli and germ cell population dysfunction in adult Caucasian men with non-mosaic Klinefelter syndrome and could help us to predict negative outcomes of SR at TESE with 100 % sensitivity when serum levels of AMH are below 2.5 pmol/l.

**Supplementary Information:**

The online version contains supplementary material available at 10.1186/s12610-021-00133-9.

## Résumé

**Introduction:**

L’extraction chirurgicale de spermatozoïdes testiculaires (ECST) est la méthode qui permet d’offrir aux hommes ayant une azoospermie des chances de paternité via l’assistance médicale à la procréation. Cependant, le manque de biomarqueurs fiables rend impossible de prédire les résultats de l’ECST. À ce jour, peu d’attention a été accordée aux valeurs sériques d’hormone anti-müllérienne (AMH) chez les hommes adultes ayant une spermatogenèse altérée. Dans cette étude, nous avons cherché à déterminer si les concentrations sériques d’AMH et le rapport AMH sur testostérone totale (AMH/T) pouvaient être des facteurs prédictifs des résultats de l’ECST dans une cohorte de 155 hommes adultes caucasiens ayant une azoospermie.

**Résultats:**

Les concentrations sériques d’AMH étaient significativement plus faibles dans l’azoospermie non-obstructive (ANO) non inexpliquée, ANO associée à un antécédent de cryptorchidie, ANO d’origine cytotoxique et génétique (médianes [pmol/l] = 30,1; 21,8; 26,7; 7,3; et *p* = 0,02; 0,001; 0,04; <0,0001, respectivement) comparativement au groupe contrôle d’azoospermie obstructive (AO) (médiane = 44,8 pmol/l). Les plus faibles valeurs ont été observées dans le groupe d’ANO d’origine génétique (*p* = 0,0001, par rapport à l’ANO non inexpliquée) et particulièrement chez les individus avec un syndrome de Klinefelter (médiane = 2,3 pmol/l, *p* <0,0001).

Seules les concentrations sériques d’AMH différaient significativement entre les individus avec résultats positifs et négatifs d’extraction de spermatozoïdes chez les hommes atteints d’un syndrome de Klinefelter non mosaïque. Un seuil optimal du taux sérique d’AMH a été fixé à 2,5 pmol/l. La sensibilité, la spécificité, la valeur prédictive positive, la valeur prédictive négative et l’exactitude de ce seuil pour prédire un résultat négatif étaient de 100 %, 76,9 %, 66,6 %, 100 % et 84,2 %, respectivement.

**Conclusions:**

Seules les concentrations sérique d’AMH, et non pas le rapport AMH/T, sont un bon marqueur du dysfonctionnement des cellules de Sertoli ainsi que des cellules germinales chez les hommes adultes caucasiens atteints du syndrome de Klinefelter non mosaïque. Elles peuvent prédire un résultat négatif du prélèvement de spermatozoïdes lors de l’ECST avec une sensibilité de 100 % lorsque les niveaux sériques sont inférieurs à 2,5 pmol/l.

## Background

Azoospermia is a major cause of male infertility, with a prevalence of about 1 % in the male population. It is defined by the complete absence of spermatozoa in the ejaculate [1].

Azoospermia can be divided into two main categories: (1) mechanical obstruction along the seminal tract [obstructive azoospermia (OA)], which may be congenital or caused by trauma (surgical or non-surgical) or infection; and (2) intrinsic testicular impairment of sperm production [nonobstructive azoospermia (NOA)]. Spermatogenesis is largely fully preserved in OA. NOA is diagnosed in 60 % of azoospermic men [2].

Many studies on NOA have reported overall success in retrieval rates, ranging from 30 to 60 %, which means that 30–60 % of individuals with NOA have focal areas of sperm production within the testes [3–5]. Testicular sperm extraction (TESE) combined with intracytoplasmic sperm injection (ICSI) have offered men diagnosed with NOA the possibility of fathering children with their own spermatozoa.

TESE is an invasive procedure, however, which exposes patients to potential testicular complications such as hematoma, inflammation, permanent devascularization and fibrosis. Moreover, an unsuccessful sperm recovery procedure in azoospermic patients has significant emotional and financial implications. Non-invasive objective markers of focal spermatogenesis would therefore be useful for distinguishing patients with a good chance of sperm retrieval from patients who have a poor chance. Although numerous studies have explored this topic, there is still debate regarding the existence or absence of predictive objective markers [6]. Collectively, these findings suggest a failure to identify parameters with reliable predictive values that could provide enough information on the routine clinical practice before TESE or micro-TESE.

Anti-Müllerian hormone (AMH), also known as Müllerian-inhibiting substance, is a homodimeric glycoprotein that belongs to the transforming growth factor (TGF)-β family. AMH is the first hormone secreted by immature Sertoli cells and is responsible for the regression of Müllerian ducts in the male fetus as part of the sexual differentiation process [7, 8]. Its role is thereafter less clear, however. Serum AMH has diagnostic applications in pediatric endocrinology as a specific marker of immature Sertoli cell number and function during childhood [9]. AMH expression is under the positive control of FSH and the inhibitory paracrine action of testosterone [10]; To date, little attention has been given to AMH serum levels in adult men with altered spermatogenesis. As a result, primary testicular failure can be reflected by an alteration in the serum levels of testicular hormones such as AMH and testosterone, and using the ratio of AMH to total testosterone (AMH/T) can offer a potentially effective biomarker for predicting the severity of testicular failure, as has been recently reported [11].

In the present study, we aimed to investigate whether serum concentrations of AMH and AMH/T values might be predictive factors for sperm retrieval outcomes during TESE in adult Caucasian men with azoospermia.

## Patients and methods

### Study design

A retrospective study was conducted in Lille University Hospital by recruiting patients seen at the Department of Andrology between January 1, 2017 and December 30, 2019 for azoospermia and who had undergone TESE.

This study followed the tenants of the Declaration of Helsinki and was approved by the Ethics Committee of the Lille University Hospital (No. DEC19-466). All patients signed an informed consent agreeing to provide their own anonymous information and tissue specimens for future studies.

### Inclusion and exclusion criteria

#### Inclusion criteria

Included patients were those with azoospermia confirmed by at least two consecutive semen analyses according to the WHO criteria [1], undergoing conventional TESE, and who received a hormonal assessment including blood assay for AMH prior to surgery, and were found to be free from any known viral or bacterial infections at the time of TESE and throughout the preceding 6 months.

#### Exclusion criteria

Patients were excluded if any of the following were present: use of hormonal preparations in the last 3 months, a known hypothalamic or pituitary defect, previous vasectomy, testosterone replacement therapy, treatments that may affect spermatogenesis, or previous tumors, including testicular tumors, not treated by chemotherapy or radiotherapy before the TESE.

### Patients and study outcomes

Complete data [clinical characteristics, hormonal profiles, seminal markers, genetic analysis, transrectal ultrasound (TRUS), ultrasound of the testes and sperm retrieval outcomes at surgery] from 155 men with azoospermia were analyzed.

**Fig. 1:**
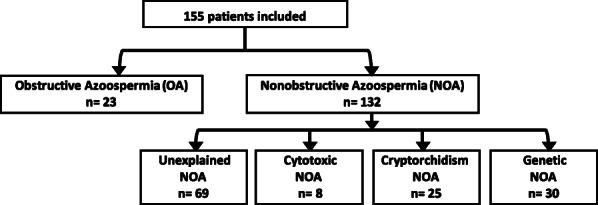
Flow chart of the study. 155 adult men with azoospermia who had undergone testicular sperm extraction were included. NOA *Nonobstructive azoospermia*, OA *Obstructive azoospermia*

The flow chart of the study is presented in Fig. 1.

Every patient underwent a detailed examination to identify the etiology of azoospermia, including a complete history, physical examination, assessment of seminal markers (including measurements of seminal plasma levels of fructose, acid phosphatase, citric acid, and α-glucosidase), ultrasound of the testes (mean testicular volume) and TRUS, and hormone profiles [measurements of serum follicle-stimulating hormone (FSH), luteinizing hormone (LH), AMH, inhibin B, and total testosterone (T)]. Genetic evaluations including chromosome karyotyping and research of Y chromosome microdeletions were performed. Hormonal assays and the genetic analysis were carried out in the same laboratories for all patients. Testicular ultrasounds were performed by the same physician.

### Etiological classification

The etiology of the azoospermia was defined from the clinical and ultrasound records. Consequently, patients were assigned to the either the obstructive or nonobstructive azoospermia etiological groups.

#### Obstructive azoospermia group (*n* = 23)

Seminal duct obstruction was assessed by clinical findings, hormonal profile, seminal biochemistry, echography and direct observation during surgery. Twenty patients presented congenital absence of the vas deferens based on physical examination, absence of seminal vesicles on echography and cystic fibrosis transmembrane conductance regulator (CFTR) gene mutations. One patient had post-infectious epididymal obstruction based on a history of acute epididymitis, epididymal dilatation on echography and low seminal alpha-glucosidase. In two cases, bilateral aplasia of the epididymis was assessed by echography and confirmed during surgical exploration.

#### Nonobstructive azoospermia group (*n* = 132)

Spermatogenesis failure was evaluated by testicular hypotrophy and/or elevated FSH levels. Bilateral testicular hypotrophy was defined as a testicular volume of 15 mL or less in both testes [12]. NOA was defined as azoospermia without any sign of obstruction and with a serum FSH level > 7.8 IU/L or a testicular volume < 15 ml [12, 13]. This group was subdivided into the following categories according to the etiology of spermatogenic failure.

#### Genetic spermatogenic failure (*n* = 30)

Nineteen individuals had non-mosaic Klinefelter syndrome (47,XXY), nine had AZF microdeletions (seven AZFc microdeletions; two AZF b and c microdeletions), one had translocation (20;22)(q11.2;p11.1) [14], and one had inversion (1)(p22q44) [15].

#### Cryptorchidism-related spermatogenic failure (*n* = 25)

Cryptorchidism was bilateral in 10 cases and unilateral in 15 individuals. An orchidopexy was performed in 19 cases. The median age at orchidopexy was 5 years. Six cases were not treated, with testes that were hardly palpable in the inguinal position.

#### Cytotoxic spermatogenic failure (*n* = 8)

This occurred in cases of chemotherapy for Hodgkin disease (*n* = 2), neuroblastoma (*n* = 1), germ cell tumor (*n* = 1), rectal cancer with radiotherapy (*n* = 1), and in three cases of leukemia treated with chemotherapy followed by radiotherapy and bone marrow transplant.

Unexplained NOA (*n* = 69) when no etiological factors could be identified.

### Hormone analysis

All hormone assays were performed on serum samples in the same laboratory and at the same time (9 a.m.) of day. Venous blood samples for hormone assays were obtained prior to the TESE.

Fully automated sandwich chemiluminescent immunoassay using the DXl Access system (Beckman Coulter, USA) was used to measure serum AMH levels. Its limit of quantification was 0.47 pmol/L. Below this threshold, AMH was defined as undetectable. Intra- and inter-assay coefficients of variation were < 2 and < 3 %, respectively.

FSH, LH and T were measured by immunoassay using an automatic analyzer (Architect, Abbott Laboratories, USA). The limit of quantification of each assay was as follows: FSH = 0.2 UI/L; LH = 0.5 UI/L, and T = 0.04 ng/ml. Intra- and inter-assay coefficients of variation were 3.1 to 5.6 % for FSH, 4.3 to 6.4 % for LH, and 4.7 to 5.2 % for T [16].

Serum inhibin B assay was determined using the Inhibin B Gen II ELISA kit (Beckman Coulter, Villepinte, France). The assay’s limit of detection was 8 pg/ml. Below this threshold, inhibin B was defined as undetectable.

When a hormone serum level was undetectable, it was assigned the value of the limit of quantification. This was the case for inhibin B in 36 cases and for AMH in three cases.

### Testicular biopsy and histologic analysis

All men with NOA underwent TESE under general anesthesia. Open TESE was performed in a single site for each testis with a transverse scrotectomy. Once the testis was delivered and inspected, a transverse incision of about 5 mm through the tunica albuginea was made with a #15 blade scalpel. The testis was then gently squeezed and the seminiferous tubules that protruded were excised with iris scissors. At the time of testicular biopsy, the parenchyma was immediately immersed in culture medium (Ferticult; FertiPro, Beernem, Belgium) in a petri dish (BD Biosciences; Erembodegem, Belgium) and sent to the reproductive biology laboratory for sperm extraction.

Among individuals with NOA, 17/132 (12.9 %) had a unilateral TESE and 115/132 (87.1 %) had bilateral TESE. TESE was performed by three surgeons with over 10 years’ experience in the field.

Individuals with OA first underwent microsurgical epididymal sperm aspiration (MESA), and in cases of failure to retrieve motile sperm (*n* = 3/23) with MESA, patients underwent unilateral TESE.

### Fresh testis examination

Sperm extraction was performed by mincing and shredding the testicular tissue in culture medium with two scalpels in order to dilacerate the seminiferous tubules. The resulting suspension was calibrated in multiple droplets under oil and incubated at 37 °C in 5 % CO2. An aliquot of each sample was examined under an inverted microscope with Hoffman optics (Leica DM IRB, Leica Microsystems; Wetzlar, Germany) at a total magnification of 400x. When spermatozoa were found, their number and vitality were rated. TESE was considered successful if at least one spermatozoon was observed in the biopsy. From our experience in the freezing-thawing procedure of testicular sperm, the suspension was frozen if a least more than one motile or immotile but alive sperm (using a hypo-osmotic swelling test) was detected in 10 µl. The suspension cell was then loaded into straws and frozen for further use in ICSI cycles. TESE outcomes were also read by two physicians with over 10 years’ experience in the field.

### Statistical analysis

In all analyses, the normality of studied variables distribution was assessed using histograms and the Shapiro-Wilk test. Comparisons between two variables were made using the Student’s t-test for Gaussian continuous variables, the Mann-Whitney U test for non-Gaussian continuous variables and the Chi-squared test (or Fisher’s exact test for expected cell frequency < 5) for categorical variables, as appropriate. Comparisons between more than two variables were made using the one-way ANOVA test for Gaussian continuous variables and the Kruskal-Wallis test for non-Gaussian continuous variables. The two-stage linear step-up procedure of Benjamini, Krieger and Yekutieli post hoc tests was used. The relationships between the quantitative data were studied by Spearman’s correlation. Receiver Operating Characteristic (ROC) curve was obtained to quantify the predictive accuracy (Area Under the Curve, AUC) of AMH serum levels [17].

All tests were two-tailed, with a significance level set at 0.05. All calculations were carried out using GraphPad Prism 7.0 software (La Jolla, California, USA).

## Results

### Cohort characteristics

The andrological phenotype characteristics of the 155 men, stratified according to the azoospermia etiology, are summarized in Table 1. The median age of the study population at the TESE procedure was 33 years. The median age of individuals with OA and NOA were 30 years and 33 years, respectively (*p* = 0.008).

**Table 1:** Comparison between clinical and hormonal profiles (whole cohort, *n* = 155) according to the etiology of azoospermia

	OA *n* = 23	Unexplained NOA *n* = 69	Cryptorchidism NOA *n* = 25	Cytotoxic NOA *n* = 8	Genetic NOA *n* = 30
Age at TESE (*years*) [median (IQR)]	30 (28–35)	34 (32-37.5)	33 (31–37)	28.5 (26.5–42.5)	32.5 (29–37)
*P*-value	-	*0.01	ns	ns	ns
Mean testicular volume (*mL*) [median (IQR)]	19.5 (15.1–22)	9 (6.5–13.4)	7 (6.4–9.7)	5.6 (3.5;11)	3.5 (2.3–7.4)
*P*-value	-	*<0.0001	*<0.0001	*<0.0001	*<0.0001 **<0.0001 ***0.01
FSH (IU /L) [median (IQR)]	3.1 (1.8–3.8)	17.2 (13.3–21.5)	23.3 (15.4–30)	17.3 (9.7–32.4)	24.8 (17.8–31.5)
*P*-value		*<0.0001	*<0.0001	*0.0002	*<0.0001 **0.01
LH (IU /L) [median (IQR)]	2.9 (2-3.7)	6.3 (4.9–8.3)	5.3 (4.6–9.7)	7.2 (6.5–9.7)	9.8 (4.7–13.8)
*P*-value		*<0.0001	*<0.0001	*<0.0001	*<0.0001 **0.04
Testosterone (ng/ml) [median (IQR)]	5.5 (4.2–7.1)	4.3 (3.5-5)	3.9 (2.4–4.8)	4.5 (2.4–6.8)	3.2 (2-4.7)
*P*-value		*0.009	*0.0007	ns	*<0.0001 **0.02
AMH (pmol/l) [median (IQR)]	44.8 (36-72.3)	30.1 (15.2–58.5)	21.8 (13.2–35.1)	26.7 (10.4–41.6)	7.3 (1.7–24.4)
*P*-value		*0.02	*0.001	*0.04	*<0.0001 **<0.0001 ***0.04
AMH/Testosterone [median (IQR)]	1.1 (0.7–1.6)	0.9 (0.5–1.9)	0.7 (0.6–1.3)	0.6 (0.4–1.3)	0.2 (0.1–0.7)
*P*-value		ns	ns	ns	*<0.0001 **<0.0001 ***0.005
Inhibin B (pg/ml) [median (IQR)]	166 (137–244)	17 (10-32.5)	11 (8-50.5)	9.5 (9–38)	9.5 (8-20.5)
*P*-value		*<0.0001	*<0.0001	*<0.0001	*<0.0001 **0.03

Statistical analysis was performed by Kruskal-Wallis test with the two-stage linear step-up procedure of Benjamini, Krieger and Yekutieli post hoc tests

*IQR* Interquartile range (first and third quartiles), *NOA* Nonobstructive azoospermia, *OA* Obstructive azoospermia, *ns* non-significant, *TESE* Testicular sperm extraction

* comparison between OA and NOA subgroups, ** comparison between unexplained and genetic NOA, *** comparison between cryptorchidism and genetic NOAg

### Hormonal profiles

**Fig. 2:**
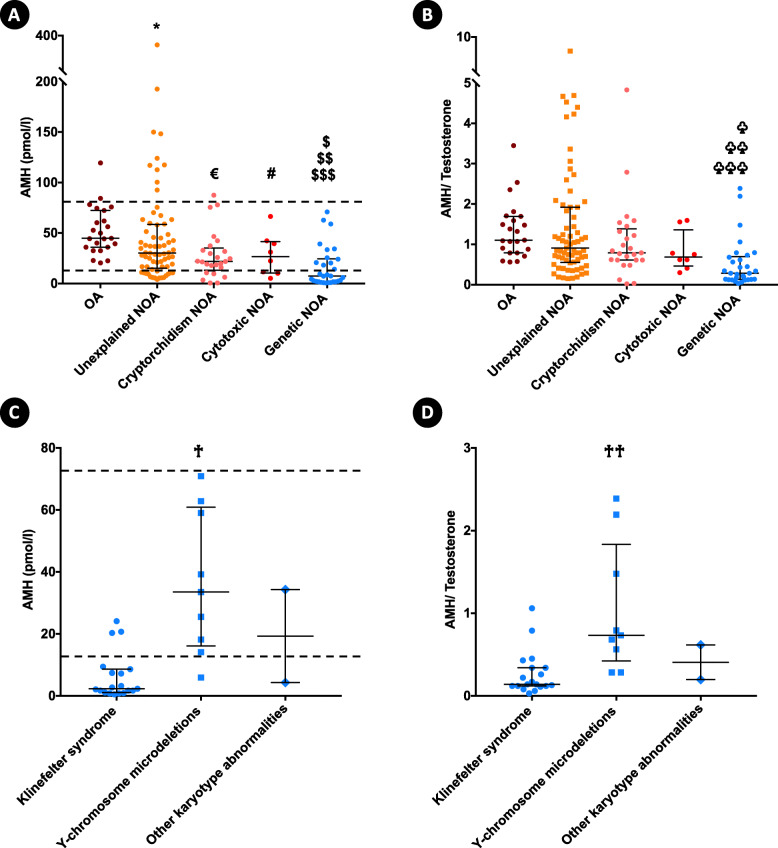
**a** Anti-Müllerian hormone (AMH) serum levels and **b** AMH to total testosterone (AMH/T) values in azoospermia according to etiology. For AMH serum levels:* (*p*=0.02) unexplained nonobstructive azoospermia (NOA) versus obstructive azoospermia (OA), **€** (*p*=0.001) cryptorchidism-related spermatogenic failure (Cryptorchidism) NOA versus OA, **# **(*p*=0.04) cytotoxic spermatogenic failure (Cytotoxic) NOA versus OA, **$ **(*p*<0.0001) genetic spermatogenic failure (Genetic) NOA versus OA, **$$ **(*p*<0.0001) Genetic versus unexplained NOA. **$$$ **(*p*=0.04) Cryptorchidism NOA versus Genetic NOA. ***For AMH/T:*** **♧** (*p*<0.0001) Genetic NOA versus OA, **♧ ♧ **(*p*<0.0001) Genetic versus unexplained NOA. **♧ ♧ ♧ **(*p*=0.005) Cryptorchidism NOA versus Genetic NOA. Statistical analysis was performed by the Kruskal-Wallis test with the two-stage linear step-up procedure of Benjamini, Krieger and Yekutieli post hoc tests. **c** and **d** Anti-Müllerian hormone (AMH) serum levels and AMH to testosterone ratio values in different categories of genetic NOA. **♰** (*p*=0.0001) Klinefelter syndrome NOA versus Y-chromosome microdeletions NOA. **♰♰** (*p*=0.0005) Klinefelter syndrome NOA versus Y-chromosome microdeletions NOA. Statistical analysis was performed by the two-tailed Mann Whitney U test. Horizontal bars show median values and interquartile ranges. Dashed lines represent the reference values range for the serum level of AMH [14–78.5 pmol/l]. AMH/*Testosterone AMH-to-total testosterone ratio*, NOA *Nonobstructive azoospermia, *OA *Obstructiveazoospermia*

Hormonal profiles according to the etiology of azoospermia are summarized in Table 1. AMH serum levels and AMH/T values were graphically represented according to the etiology of azoospermia in Fig. 2a and b, and for individuals with different categories of genetic NOA in Fig. 2c and d.

A large majority of AMH serum values for individuals with OA were in the reference values range for fertile men [14–78.5 pmol/l], and the sperm retrieval rate was 100 %. These were the reasons that a group of individuals with OA was considered as a control group.

AMH serum levels were significantly lower in unexplained, cryptorchidism-related, cytotoxic and genetic NOA compared with obstructive azoospermia. The lowest values were observed in cases of genetic NOA ([*p* < 0.0001] compared with unexplained NOA) and especially in individuals with non-mosaic Klinefelter syndrome (*p* = 0.0001 ). Phenotypic characteristics of individuals with non-mosaic Klinefelter syndrome are summarized in Table 2.

**Table 2:** Phenotypic characteristics of men with NOA related to Klinefelter syndrome (*n* = 19) according to the testicular sperm extraction outcomes

	Overall Klinefelter syndrome individuals *n* = 19	Positive sperm retrieval *n* = 6	Negative sperm retrieval *n* = 13	*P*-value
Age at TESE (years) ¤ [median (IQR)]	30 (28–34)	29.5 (24-34.2)	30 (28–35)	0.63
Mean testicular volume (mL) ¤ [median (IQR)]	2.7 (2-3.5)	3.1 (1.9–4.3)	2.5 (2-3.3)	0.21
FSH (IU /L) ¤ [median (IQR)]	30.5 (18.9–36.7)	29.9 (18.3–34.8)	30.9 (21.8–36.9)	0.61
LH (IU /L) ¤ [median (IQR)]	12.4 (9.8–15.9)	11.7 (9.4–15.2)	12.4 (9.8–17)	0.75
Testosterone (ng/ml) ¤¤ [median (IQR)]	2.6 (1.7–3.6)	3 (2.8–4.5)	1.8 (1.2–3.6)	0.14
AMH (pmol/l) ¤¤ [median (IQR)]	2.3 (1.1-8.6)	8 (3–13)	1.7 (0.8–4.7)	**0.01**
AMH/Testosterone ¤¤ [median (IQR)]	0.14 (0.1–0.3)	0.3 (0.1–0.4)	0.1 (0.08–0.2)	0.06
Inhibin B (pg/ml) ¤¤ [median (IQR)]	8 (8–10)	8 (8–14)	8 (8–10)	0.60

Statistical analysis was performed by the two-tailed Student’s t (¤) or Mann Whitney U (¤¤) tests

*IQR* Interquartile range (first and third quartiles), *NOA* Nonobstructive azoospermia

### Correlations

Correlations between AMH and inhibin B, FSH, LH, T serum levels, and average testicular volume in each category of azoospermia are summarized in Table 3. AMH serum levels were correlated positively with both serum inhibin B and T in the unexplained NOA, cryptorchidism-related NOA and genetic NOA groups. Serum concentrations of AMH were negatively correlated with LH serum levels in the unexplained and genetic NOA groups. AMH serum levels were only correlated negatively with serum concentrations of FSH in individuals with genetic NOA.

**Table 3:** Correlations between AMH and inhibin B, FSH, LH, total testosterone serum levels, mean testicular volume and age at TESE in each category of azoospermia

		OA *n* = 23	Unexplained NOA *n* = 69	Cryptorchidism NOA *n* = 25	Cytotoxic NOA *n* = 8	Genetic NOA *n* = 30
Mean testicular volume (ml)	*r*	0.50	0.39	0.52	0.09	0.71
	*P*-value	**0.01**	**0.0008**	**0.006**	0.82	**< 0.0001**
FSH (IU/L)	*r*	0.25	-0.21	-0.35	-0.23	-0.43
	*P*-value	0.23	0.07	0.07	0.53	**0.01**
LH (IU/L)	*r*	− 0.07	-0.24	-0.33	-0.11	-0.73
	*P*-value	0.73	**0.04**	0.09	0.78	**< 0.0001**
Testosterone (ng/ml)	*r*	0.27	0.36	0.47	0.70	0.76
	*P*-value	0.19	**0.002**	**0.01**	**0.04**	**< 0.0001**
Inhibin B (pg/ml)	*r*	0.36	0.27	0.45	0.72	0.67
	*P*-value	0.08	**0.02**	**0.02**	0.05	**< 0,0001**

The relationship between AMH serum levels and other hormonal profiles and mean testicular volume was assessed by Spearman’s correlation

*IQR* Interquartile range (first and third quartiles), *NOA* Nonobstructive azoospermia, *OA* Obstructive azoospermia, *r* Spearman’s coefficient of correlation, *TESE* Testicular sperm extraction

### Sperm retrieval rate and predictive factors of TESE outcomes

The best sperm retrieval rate was obtained in the OA group (100 %). Among the NOA groups, cryptorchidism-related NOA had the best rate of sperm retrieval (15/25; 60 %). The lowest sperm retrieval rates were observed in cytotoxic NOA (1/8; 12.5 %) (Table 40).

**Table 4:** Distribution of individuals according to the etiology of NOA and the testicular sperm extraction outcomes

	Overall NOA individuals *n *= 132	Sperm retrieval		*P*-value
		Successful *n* = 45	Failure *n* = 87	
Etiology				
Unexplained NOA ¤	69	20 (30 %)	49 (70 %)	*0.19
Cryptorchidism NOA ¤	25	15 (60 %)	10 (40 %)	**0.006
Cytotoxic NOA ¤¤	8	1 (12.5 %)	7 (87.5 %)	**0.43
Hodgkin disease	2	0	2	
Leukemia	3	0	3	
Neuroblastoma	1	0	1	
Germ cell tumor	1	0	1	
Rectal cancer	1	1	0	
Genetic NOA ¤	30	9 (30 %)	21 (70 %)	**0.91
* Klinefelter syndrome*	19	6	13	
* Other karyotype abnormalities*	2	0	2	
* Y-chromosome microdeletions*	9	3	6	

Statistical analysis was performed by the two-tailed Chi-squared (¤) or Fisher’s exact (¤¤) tests

*Abbreviations*: *OA* Obstructive azoospermia, *NOA* Nonobstructive azoospermia,*TESE* Testicular sperm extraction

(*) versus other etiologies of NOA, (**) versus unexplained NOA

### Predictive factors associated with successful sperm retrieval

The comparisons between the clinical and hormonal profiles of positive and negative sperm retrieval outcomes in unexplained, cryptorchidism-related and genetic NOA groups are summarized in Tables 1, 2 and 3 in the Supplementary Data.

**Fig. 3:**
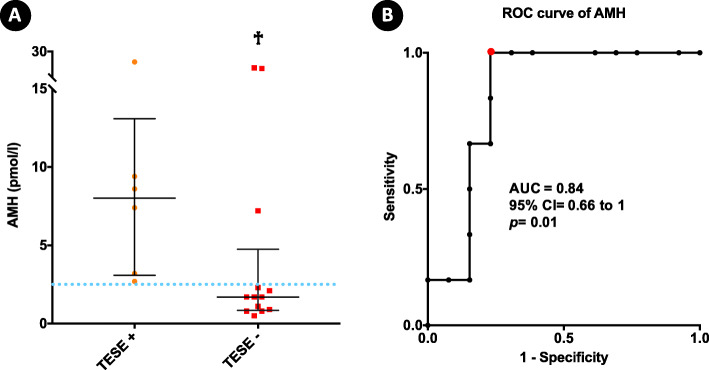
Circulating AMH serum levels were predictive of negative sperm retrieval outcomes in individuals with Klinefelter syndrome-related spermatogenic failure undergoing testicular sperm extraction (TESE).** a** Dot plots depict values from the 6 positive sperm retrievals and 13 negative sperm retrievals in Klinefelter syndrome NOA; horizontal bars detail median values and interquartile ranges. Dashed lines represent the cut-off chosen for AMH. **♰** (*p*=0.01) positive versus negative TESE in individuals with Klinefelter syndrome NOA. Statistical analysis was performed by the two-tailed Mann Whitney U test. **b **ROC-derived curve showing the AUC, 95% CI (confidence intervals) and P-value. Red dot plots depict optimal AMH cut-off (2.5 pmol/l). Area under the ROC curve (AUC) = 0.84; (95% CI = 0.66 to 1; *p*= 0.01). For the AMH cut-off: sensitivity, specificity, positive predictive value (PPV), negative predictive value (NPV) and accuracy: 100%, 76.9%, 66.6%, 100% and 84.2%, respectively (*p*=0.003). AUC *Area Under the Curve,*95% CI*95% confidence interval*,NOA *Nonobstructive azoospermia*, ROC *Receiver operating characteristic*, TESE *Testicular sperm extrac*

Median values of the serum AMH or AMH/T were not significantly different between the positive and negative sperm retrieval groups in individuals with unexplained, cryptorchidism-related and genetic NOA. However, significantly lower median values of serum AMH were observed between the negative (median [IQR]: 1.7 [0.8; 4.7]) and positive (median [IQR]: 8 [3; 13]) sperm retrieval groups for individuals with NOA related to non-mosaic Klinefelter syndrome (*p* = 0.01) (Fig. 3a; Table 4).

### Receiver operating characteristic curve of AMH based on sperm retrieval outcomes

Since the median values of serum AMH differed significantly between the positive and negative sperm retrieval outcomes in individuals with non-mosaic Klinefelter syndrome-related NOA, an ROC analysis was performed to evaluate the predictive accuracy of AMH in this group. AMH had an AUC of 0.84 (95 % CI = 0.66 to 1; *p* = 0.01). AMH serum levels were then dichotomized according to the most informative cut-off value capable of maximizing sensitivity while predicting negative sperm retrieval. The most informative cut-off value for serum AMH was set at 2.5 pmol/l. In this context, circulating serum AMH levels below 2.5 pmol/l were associated with negative sperm retrieval outcomes with 100 % sensitivity and 76.9 %, specificity, a negative predictive value of 100 % and a positive predictive value of 66.6 % (Fig. 3b).

## Discussion

Few cohort studies have investigated the serum levels of AMH in adult men with azoospermia. This prompted us to evaluate the serum concentrations of AMH in a cohort of 155 adult Caucasian men with azoospermia from different etiologies and to assess its predictive value in terms of TESE outcomes.

In our study, lower median values of AMH serum levels were observed in the cytotoxic, cryptorchidism-related, unexplained, and genetic NOA groups compared with the control group of individuals with OA. In individuals with OA, a large majority of AMH serum values were in the reference values range for fertile men (D. Chevenne, pers. comm.). Previous studies reported that no significant differences in AMH serum levels were found in cases of oligospermia and OA relative to infertile individuals with normal sperm concentrations [18, 19]; decreased serum levels of AMH were reported, however, in NOA versus controls or individuals with OA [18, 20].

Close results reported in our series were observed in the study published by Plotton et al. [21]. They reported in their short series of men with azoospermia that the lowest AMH plasma levels were observed in individuals with NOA, and especially in men with Klinefelter syndrome.

The follow-up of boys with Klinefelter syndrome throughout puberty showed that Sertoli cell function was normal until mid-puberty, when a dramatic impairment was observed with a decrease of AMH serum levels much lower than the control group [22]. Therefore, the decrease in AMH serum levels seems to be a specific feature of testicular failure associated with Klinefelter syndrome.

Current data showed that both radiotherapy and chemotherapy primarily affected germ cells of the testis, whereas Leydig cells were less affected [23, 24]. In our study, both Sertoli cell markers, AMH and inhibin B serum levels were lower in individuals with cytotoxic NOA. In addition, this was associated with a low sperm retrieval rate. A few clinical studies had been performed previously to explore the direct effects of cancer treatments on Sertoli cell function. In fact, two studies including a few patients treated with polychemotherapy or hematopoietic cell transplantation have shown that serum AMH levels were below normal for age [25, 26].

In our study, individuals with cryptorchidism NOA had lower serum AMH and inhibin B levels compared with the control group. Also, previous studies reported that individuals with unilateral or bilateral cryptorchidism had a decrease of serum AMH levels compared with controls [27, 28].

In our series, the median value of serum AMH levels was only significantly different between the positive and negative sperm retrieval outcomes in individuals with non-mosaic Klinefelter syndrome-related NOA. Therefore, using the ROC curve, we found that the values of circulating serum AMH allowed us to differentiate a cohort of adults with non-mosaic Klinefelter syndrome-related NOA into positive versus negative sperm retrieval outcomes at the TESE. Adopting a serum AMH threshold below 2.5 pmol/l resulted in 100 % sensitivity and a negative predictive value for negative sperm retrieval. Contrary to our results, Rohayemet et al. reported in a large cohort of adolescents with non-mosaic Klinefelter syndrome that AMH serum levels could not predict the success of sperm retrieval with micro-TESE [29]. In our series, the lowest values of serum AMH were observed in individuals with non-mosaic Klinefelter syndrome, which was associated with negative sperm retrieval outcomes. This could be an indicator of testis failure of exocrine functions. In other words, impaired function of Sertoli cells associated with spermatogenic failure.

Alfano et al., in a short cohort of white Caucasian individuals with unexplained NOA, showed that the median serum AMH and AMH/T values were significantly higher in men with negative sperm retrieval compared with those having positive sperm retrieval with micro-TESE. They had chosen a cut-off for AMH and AMH/T (> 33 pmol/l or > 4.62 ng/ml and > 1.02, respectively), which resulted in 100 % sensitivity for negative sperm retrieval outcomes. In addition, they hypothesized that not only unexplained NOA individuals with negative sperm retrieval expressed a testicular phenotype of immature Sertoli cells with germ cell aplasia, but also that the impaired downregulation of AMH was due to unresponsive testosterone action, like at the pre-pubertal stage [11]. Contrary to a recently published study [11], median of AMH/T values in our series were not significantly different between the positive and negative sperm retrieval outcomes in individuals with NOA.

Some previous studies reported, in a population of adult men with NOA, that AMH was negatively correlated with FSH and positively correlated with inhibin B and T serum levels [18, 21]. Similar results were observed in our study, which corroborates the idea that serum AMH could serve as a good marker for evaluating Sertoli cell alterations in adults with unexplained, cryptorchidism-related and genetic NOA. Therefore, the reduction of serum AMH levels observed in some individuals with NOA was not due to the suppressive action of androgens such as at puberty but to the impaired functioning of both Sertoli and Leydig cells.

The strengths of our study are that it includes a relatively average cohort representative of infertile male patients, with many etiologies of azoospermia studied with the outcomes of testicular sperm retrieval. To our knowledge, this is the first report to describe the usefulness of serum AMH levels for predicting negative sperm retrieval outcomes with TESE in adults with non-mosaic Klinefelter syndrome-related NOA. A major limitation is the retrospective study design, which prevents us from including patients without an AMH measurement before TESE. The average number of patients in different groups of individuals with NOA did not allow us to carry out multivariate statistical analyses. Also, an unequal number of patients between successful and failed sperm retrieval in some groups may be a limitation for the interpretation of results. In our department, as in the majority of andrology departments in France, we used conventional TESE, which may not be representative of the whole testicular histology. Some authors have reported that micro-TESE could be particularly more effective than conventional TESE in recovering sperm from men with a testicular volume of less than 10 ml [30].

As reported previously [31], ethnic origin can affect the serum circulating hormonal levels such as LH, T and free testosterone in men or AMH in women [32]. This can be explained by recent data from a large series of monozygotic and dizygotic male twins [33] and a genome-wide association study [34]. Both concluded that circulating AMH levels in adult men are under a strong genetic influence. This can be a limiting factor in comparing our results with previously published studies. As a result, a prospective study including a larger number of individuals with different NOA categories and ethnic origins is required to draw a definitive conclusion.

In conclusion, despite some limitations, our results suggest that serum AMH levels, but not AMH/T values, are a good marker for evaluating Sertoli cell alterations in unexplained, cryptorchidism-related and genetic NOA, but it cannot serve as a stand-alone marker of persistent spermatogenesis in all cases of NOA. However, serum concentrations of AMH could help us to predict the negative sperm retrieval outcomes at TESE with 100 % sensitivity and 76.9 % specificity in Caucasian adults with non-mosaic Klinefelter syndrome when serum AMH levels are below 2.5 pmol/l.

In summary unlike serum inhibin B, serum AMH levels could be a good indicator of testis failure for both exocrine and endocrine functions; this can help practitioners monitor testicular function and assist in the decision-making about the time to explore fertility or to perform TESE in young adult men with a medical or surgical history that can cause testicular damage and affect their function.

## Supplementary information

**Additional file 1.**

## Data Availability

The data sets generated and/or analyzed during the current study are available from the corresponding author upon reasonable request.

## Abbreviations

AMH: Anti-Müllerian hormone
AMH/T: AMH-to-total testosterone ratio
AUC: Area Under the Curve
CFTR: Cystic fibrosis transmembrane conductance regulator
FSH: Follicle-stimulating hormone
ICSI: Intracytoplasmic sperm injection
LH: Luteinizing hormone
Micro-TESE: Microsurgical testicular sperm extraction
NOA: Nonobstructive azoospermia
OA: Obstructive azoospermia
ROC: Receiver Operating Characteristic
T: Total testosterone
TESE: Testicular sperm extraction
TGF: Transforming growth factor
TRUS: Transrectal ultrasound
