# Supplementary material for: Contribution of serum anti-Müllerian hormone in the management of azoospermia and the prediction of testicular sperm retrieval outcomes: a study of 155 adult men
Source: Basic Clin Androl. 2021 Jun 17;31:15. doi: 10.1186/s12610-021-00133-9 (PMC8210365; doi:10.1186/s12610-021-00133-9)
Supplement: Supplementary file 1 — Additional file 1. [file 12610_2021_133_MOESM1_ESM.docx]

**Supplementary Data**

**Supplementary table 1. Phenotypic characteristics of men with unexplained NOA (n = 69) according to the testicular sperm extraction outcomes.**

Statistical analysis was performed by the two-tailed Student’s t (¤) or Mann Whitney U (¤¤) tests.

IQR *Interquartile range (first and third quartiles)*, NOA *Nonobstructive azoospermia*.

|  | Positive sperm retrieval  n= 20 | Negative sperm retrieval  n= 49 | *P*-value |
| --- | --- | --- | --- |
| Age at TESE (years) ¤¤  [median (IQR)] | 34.5 (32.2-39) | 34 (31-37) | 0.20 |
| Mean testicular volume (mL) ¤¤  [median (IQR)] | 7.1 (6-9.2) | 10.4 (6.9-13.5) | 0.05 |
| FSH (IU /L) ¤¤  [median (IQR)] | 16.3 (13.8-20.5) | 17.9 (12.9-22) | 0.69 |
| LH (IU /L) ¤¤  [median (IQR)] | 6.3 (4.9-9.3) | 6.3 (4.9-8.3) | 0.97 |
| Testosterone (ng/ml) ¤  [median (IQR)] | 4.3 (3.5-4.7) | 4.4 (3.4-5.2) | 0.25 |
| AMH (pmol/l) ¤¤  [median (IQR)] | 26 (10.1-55.4) | 33.3 (17.9-58.5) | 0.17 |
| AMH/Testosterone ¤¤  [median (IQR)] | 0.94 (0.39-1.7) | 0.91 (0.64-2.1) | 0.31 |
| Inhibin B (pg/ml) ¤¤  [median (IQR)] | 17 (10-31.2) | 18 (10-33.5) | 0.92 |

**Supplementary table 2. Phenotypic characteristics of men with cryptorchidism NOA (n = 25) according to the testicular sperm extraction outcomes.**

Statistical analysis was performed by the two-tailed Student’s t (¤) or Mann Whitney U (¤¤) tests.

IQR *Interquartile range (first and third quartiles)*, NOA *Nonobstructive azoospermia*.

|  | Positive sperm retrieval  n= 15 | Negative sperm retrieval  n= 10 | *P*-value |
| --- | --- | --- | --- |
| Age at TESE (years) ¤  [median (IQR)] | 33  (32-37) | 33  (27.7-37.2) | 0.35 |
| Mean testicular volume (mL) ¤¤  [median (IQR)] | 7  (6.8-9.9) | 5.8  (2.8;10) | 0.25 |
| FSH (IU /L) ¤  [median (IQR)] | 18.9  (6.3-29.4) | 25  (18.6-35.8) | 0.25 |
| LH (IU /L) ¤¤  [median (IQR)] | 4.9  (2.8-9) | 7.1  (5.1-12.4) | 0.05 |
| Testosterone (ng/ml) ¤  [median (IQR)] | 4.3  (2.4-5) | 3.1  (2.3-4.8) | 0.62 |
| AMH (pmol/l) ¤¤  [median (IQR)] | 21.8  (15.9-42.4) | 22.1  (4.7-30.1) | 0.59 |
| AMH/Testosterone ¤¤  [median (IQR)] | 0.84  (0.61-1.54) | 0.73  (0.36-1.2) | 0.37 |
| Inhibin B (pg/ml) ¤¤  [median (IQR)] | 17  (8-61) | 10.5  (8-27.7) | 0.70 |

**Supplementary table 3. Phenotypic characteristics of men with genetic NOA (n= 30) according to the testicular sperm extraction outcomes.**

|  | Positive sperm retrieval  n= 9 | Negative sperm retrieval  n= 21 | *P*-value |
| --- | --- | --- | --- |
| Age at TESE (years) ¤  [median (IQR)] | 33  (26.5-34.5) | 32  (29-38.5) | 0.38 |
| Mean testicular volume (mL) ¤¤  [median (IQR)] | 4.2  (2.3-9.6) | 3.3  (2.2-6.5) | 0.69 |
| FSH (IU /L) ¤  [median (IQR)] | 24.8  (16.7-32.2) | 24.8  (17.6-32.2) | 0.88 |
| LH (IU /L) ¤  [median (IQR)] | 9.8  (5.3-14.3) | 10.7  (4.6-14) | 0.89 |
| Testosterone (ng/ml) ¤¤  [median (IQR)] | 3.5  (3-6.7) | 3  (1.7-4.3) | 0.18 |
| AMH (pmol/l) ¤¤  [median (IQR)] | 9.4  (5.3-28.8) | 4.3  (1.4-23.1) | 0.16 |
| AMH/Testosterone ¤¤  [median (IQR)] | 0.3  (0.2-0.6) | 0.2  (0.1-0.7) | 0.29 |
| Inhibin B (pg/ml) ¤¤  [median (IQR)] | 8  (8-48.5) | 10  (8-19) | 0.96 |

Statistical analysis was performed by the two-tailed Student’s t (¤) or Mann Whitney U (¤¤) tests.

IQR *Interquartile range (first and third quartiles)*, NOA *Nonobstructive azoospermia*.
